# Outcome of arthroscopic triple release combined with rotator cuff repair in the treatment of rotator cuff injury combined with frozen shoulder

**DOI:** 10.12669/pjms.40.3.7709

**Published:** 2024

**Authors:** Jieliang Hu, Kongxing Wei, Yinping Xu, Liyuan Chen

**Affiliations:** 1 Jieliang Hu, Second Department of Orthopedics, The Third Affiliated Hospital of Gansu University of Traditional, Chinese Medicine (Baiyin City First People’s Hospital), Baiyin 730900, Gansu, China; 2 Kongxing Wei, Second Department of Orthopedics, The Third Affiliated Hospital of Gansu University of Traditional, Chinese Medicine (Baiyin City First People’s Hospital), Baiyin 730900, Gansu, China; 3 Yinping Xu, Second Department of Orthopedics, The Third Affiliated Hospital of Gansu University of Traditional, Chinese Medicine (Baiyin City First People’s Hospital), Baiyin 730900, Gansu, China; 4 Liyuan Chen, Second Department of Orthopedics, The Third Affiliated Hospital of Gansu University of Traditional, Chinese Medicine (Baiyin City First People’s Hospital), Baiyin 730900, Gansu, China

**Keywords:** Arthroscopic triple release, Rotator cuff repair, Rotator cuff injury, Frozen shoulder, Pain media, Muscle strength

## Abstract

**Objective:**

To investigate the effect of arthroscopic triple release combined with rotator cuff repair in the treatment of rotator cuff injury combined with frozen shoulder and its influence on the range of motion and pain score of shoulder joint, and the levels of serum pain mediators.

**Methods:**

This was prospective study. A total of 132 patients with rotator cuff injury combined with frozen shoulder admitted to The Third Affiliated Hospital of Gansu University of Traditional Chinese Medicine from December 2020 to December 2022 were prospectively selected and divided into two groups according to the random number table method: control group (n=67) and observation group (n=65). Patients in the control group were treated with arthroscopic rotator cuff repair alone, while those in the observation group were treated with arthroscopic triple release combined with rotator cuff repair, and the surgical effects of the two groups were compared.

**Results:**

Three months after treatment, the external rotation, internal rotation, abduction, forward flexion, β-endorphin(β-EP), prostagranin E2 (PGE2) and substance P(SP)in the observation group were better than those in the control group (P<0.05), while the weight-bearing strength of the affected limb in internal rotation, external rotation and forward flexion was higher than that of the control group(P<0.05). Meanwhile, the Visual Analogue Scale (VAS) score of the observation group was lower than that of the control group at one month and three months after treatment, while the University of California at Los Angeles shoulder rating scale (UCLA) score and Constant-Murley Score (CMS) were higher than those of the control group (P< 0.05).

**Conclusion:**

Arthroscopic triple release combined with rotator cuff repair improves various effects for patients with rotator cuff injury combined with frozen shoulder, such as ameliorating the muscle strength of the affected limb and improving the level of pain mediators.

## INTRODUCTION

The rotator cuff, as an essential structure for performing shoulder joint functions, may cause limited shoulder movement and obvious pain once it is damaged. In case of long-term development, shoulder joint adhesion may be caused, affecting daily life and shoulder joint function.^1^ Frozen shoulder is primary adhesive capsulitis that can be attributed to various factors such as shoulder joint infection, long-term immobilization of the shoulder joint, and upper limb trauma.^2^ Most frozen shoulders may occur concurrently with rotator cuff injuries, with the incidence accounting for 35% of rotator cuff injuries.^3^ Once combined, the limitation of shoulder joint movement and pain will be aggravated, and early treatment is needed to alleviate the symptoms. Currently, surgical treatment is the preferred treatment for rotator cuff injury combined with frozen shoulder.

Arthroscopic surgery is favored as one of the main treatment modality of this disease by virtue of its advantages of small trauma, clear vision and high safety. Arthroscopic rotator cuff repair is commonly used in the initial stage of the disease, which can relieve symptoms and improve shoulder motion, but with an unsatisfactory overall result.^4^,^5^ With the improvement of the surgical scheme, arthroscopic triple release combined with rotator cuff repair began to be promoted in the clinic. With this technology, shoulder joint adhesion can be fully released, shoulder joint pain and limited movement signs can be relieved to a certain extent, which promotes the recovery of shoulder muscle function.^6^

However, there are few reports about this technique in China and its efficacy is still controversial. Based on this, an in-depth analysis of the efficacy of arthroscopic triple release combined with rotator cuff repair was carried out in this study, and the advantages of this technique were further investigated by taking the range of motion and pain score of the shoulder joint as prognostic indicators.

## METHODS

This was a prospective study. 132 patients with a rotator cuff injury combined with frozen shoulder admitted to The Third Affiliated Hospital of Gansu University of Traditional Chinese Medicine from December 2020 to December 2022 were prospectively selected as the study subjects.

### Ethical Approval:

The study was approved by the Institutional Ethics Committee of the Third Affiliated Hospital of Gansu University of Traditional Chinese Medicine (No.: 21CX6FD163; date: December 03, 2021), and written informed consent was obtained from all participants.

### Inclusion criteria:

- Patients who showed rotator cuff injury by imaging examination and mainly restricted external rotation, with shoulder abduction ≤90°, forward flexion ≤90°, and lateral external rotation ≤35°.
- Patients with supraspinatus tendon injury.
- Patients who signed written informed consent and participated in studies that met the ethical review of the Declaration of Helsinki.
- Patients with surgical indications.

### Exclusion criteria:

- Patients with a history of shoulder joint surgery or shoulder dislocation.
- Patients with osteonecrosis and shoulder joint tumors.
- Patients with glenoid fractures and rotator cuff tendon fractures.
- Patients with a huge rotator cuff tear with a range> 5 cm.
- Patients with rotator cuff injury with a clear history of trauma.
- Patients with severe osteoporosis.

Patients in the control group underwent arthroscopic rotator cuff repair alone. After the surgical instruments were inserted, the specific conditions of the acromion and glenohumeral joint were explored, and the instruments were inserted into the gap to clear the subacromial bursa. The rotator cuff stump was pulled with grasping forceps to make the footprints of the humeral tubercle and the rotator cuff close to each other, and then the rotator cuff insertion of the humeral tubercle was polished. If the bone surface oozes blood, stitches are required.

In contrast, patients in the observation group were treated with arthroscopic triple release combined with rotator cuff repair, and were given nerve block + low-pressure general anesthesia. The shoulder arthroscopy was inserted into the menschondral joint via the posterior approach, and an anterior approach was established. The label-articular capsule was explored sequentially, the humeral head cartilage and glenoid cavity were observed, and the subscapular tendon and the inferior surface of the rotator cuff were explored to determine the presence of adhesion, synovitis hyperplasia, and Slap injury. The reasonable tripartite release was performed according to the adhesion situation:

- The enlarged subacromial cleaning;
- The release of the glenoid labrum-ligament complex;
- The release of the subscapularis tendon in all directions to facilitate later external rotation and abduction of the shoulder joint.

After complete release, the shoulder arthroscopy was inserted into the subacromial space to remove the bursa, and a lateral approach was established to expand the subacromial space. Suture and cleanup were determined according to the extent of involvement and site of injury.

For the patients with subacromial full-thickness injury and synovial side injury, the bone bed should be made and the torn rotator cuff should be repaired by suture bridge technique or double-row or single-row sutures according to the type and size of injury; For those with lateral injury of glenohumeral joint, tendor transfixion is needed for suture and repair. Both groups are required to undergo 48 hour routine anti-infection postoperatively, with ice applied on the affected side for 48 hours, and an X-ray examination three days postoperatively. Functional exercise of the affected shoulder joint should be carried out under the guidance of physicians, and the exercise amplitude, time and frequency are supposed to be gradually strengthened.

Both groups are required to undergo 48 h routine anti-infection postoperatively, with ice applied on the affected side for 48 h, and an X-ray examination three days postoperatively. Functional exercise of the affected shoulder joint should be carried out under the guidance of physicians, and the exercise amplitude, time and frequency are supposed to be gradually strengthened. All operations were performed by the same group of doctors, which has been described in the text.

### Observation indicators:

(1) The range of motion of shoulder joint was compared between the two groups, and the external rotation, internal rotation, abduction and forward flexion of the shoulder joint were measured with a measuring instrument; (2) The muscle strength of the affected side was compared between the two groups, and the muscle strength of internal rotation, external rotation and forward flexion of the affected limb was quantitatively measured by the rapid muscle measurement system; (3) The levels of serum pain mediators were compared. Three milliliter elbow venous blood was extracted from subjects, and serum was separated at 1500 r/min for 10 min. β-endorphin (β-EP), prostagranin E2 (PGE2) and substance P(SP) were detected by radioimmunoassay. (4) The scores of each item were compared. University of California at Los Angeles shoulder rating scale (UCLA) Score.^7^ The full score is 35 points, and the assessment content includes active shoulder flexion strength, range of motion, shoulder pain, satisfaction and function. The higher the recovery of shoulder joint function, the higher the score; Visual Analogue Scale (VAS) score.^8^ The higher the score, the stronger the pain, with the highest score of 10 points; Constant-Murley Score (CMS).^9^ The highest score is 100 points, including 15 points for pain, 20 points for activities of daily living, 40 points for active joint range of motion and 25 points for muscle strength. The better the shoulder joint function, the higher the score. Mean follow up time was six months.

### Statistical Analysis:

All data were processed by SPSS21.0 statistical software, and the range of motion of the shoulder joint, muscle strength, serum pain mediator level, UCLA score, VAS score, and CMS score were in accordance with the normal distribution and expressed as (*x̅*±*S*). Repeated measurement analysis of variance was used for time-varying comparison between the two groups, and LSD-t test for pairwise comparison afterward. In general data, count data were represented by (%) and the χ^2^ test or generalized equation was used for analysis. P<0.05 indicates a statistically significant difference.

## RESULTS

There were 83 (62.88%) males and 49 (37.12%) females, with an average age of (55.32±4.45) years and an average duration of (34.78±4.13) days; Lesion site: 56 (42.42%) cases in the left shoulder and 76 (57.58%) cases in the right shoulder; Gerber typing: 81 (61.36%) cases of small and medium injuries and 51 (38.64%) cases of partial injuries. The data of the two groups are shown in Table-I, which showed no statistical difference by comparison (*P*>0.05)

**Table-I:** Comparison of general data between the two groups.

				Gender (n, %)		Lesion site (n, %)		Gerber typing (n, %)	

Group	n	Age (years old)	Course of disease (d)	Male	Female	Left shoulder	Right shoulder	Small and medium injuries	Partial injuries
Observation group	65	55.21±4.39	34.61±4.27	38 (58.46)	27 (41.54)	25 (38.46)	40 (61.54)	39 (60.00)	26 (40.00)
Control group	67	55.48±4.43	34.89±4.33	45 (67.16)	22 (32.84)	31 (46.27)	36 (53.73)	42 (62.69)	25 (37.31)
*t/χ^2^*	-	0.352	0.374	1.071		0.823		0.100	
*P*	-	0.726	0.709	0.301		0.364		0.751	

No statistical difference was observed in the comparison of the range of motion of the shoulder joint before treatment between the two groups (P>0.05). The external rotation, internal rotation, abduction and forward flexion of the observation group were better than those of the control group three months after treatment (P< 0.05). Table-II.

**Table-II:** Comparison of range of motion of shoulder joint (*χ̅*±*S*, °).

Group	n	Time	Internal rotation	External rotation	Abduction	Forward flexion
Observation group	65	Preoperatively	3.82±0.12	9.56±1.37	74.59±3.62	71.59±3.62
		3 months postoperatively	9.71±1.65	21.29±2.65	128.77±6.98	141.28±6.95
		Front-back difference	6.15±0.37	12.38±1.11	54.21±3.21	69.96±3.74
Control group	67	Preoperatively	3.59±0.34	9.72±1.42	74.68±3.51	71.68±3.51
		3 months postoperatively	7.44±0.29	15.56±1.39	105.13±4.66	120.11±5.58
		Front-back difference	4.11±0.18	6.87±1.22	31.45±2.22	49.52±4.54
*t value*	-	-	40.467	27.117	47.5	28.186
*P value*	-	-	<0.001	<0.001	<0.001	<0.001

No statistical difference was observed in the comparison of the muscle strength of the affected limbs before treatment between the two groups (P>0.05). The weight-bearing strength of internal rotation, external rotation, and forward flexion in the observation group was better than that of the control group after three months of treatment (P<0.05). Table-III.

**Table-III:** Comparison of muscle strength of affected limb (*χ̅*±*S*, kg).

Group	n	Time	Internal rotation	External rotation	Forward flexion
Observation group	65	Preoperatively	2.41±0.36	1.19±0.39	3.26±0.19
		3 months postoperatively	4.45±1.19	3.68±1.24	5.58±1.42
		Front-back difference	2.37±0.24	2.42±0.15	2.34±0.21
Control group	67	Preoperatively	2.32±0.19	1.28±0.16	3.41±0.25
		3 months postoperatively	3.52±0.13	2.27±0.28	4.38±0.28
		Front-back difference	1.17±0.11	1.21±0.10	1.15±0.23
*t value*	-	-	37.109	54.683	31.016
*P value*	-	-	<0.001	<0.001	<0.001

No statistical difference was observed in the comparison of the levels of serum pain mediums between the two groups before treatment (P>0.05). The improvement of β-EP, PGE2 and SP levels in th. Table-IV.

**Table-IV:** Comparison of the level of serum pain mediums (*χ̅*±*S*).

Group	n	Time	β-EP (ng/L)	PGE2(ng/mL)	SP(ng/L)
Observation group	65	Preoperatively	154.39±34.25	36.42±3.62	671.25±46.69
		3 months postoperatively	246.44±27.59	19.13±1.49	418.67±26.65
		Front-back difference	92.65±12.13	17.22±1.12	253.41±15.22
Control group	67	Preoperatively	154.47±34.63	36.53±3.57	671.49±46.57
		3 months postoperatively	198.13±23.42	26.69±2.28	498.55±33.29
		Front-back difference	44.51±10.22	10.49±1.12	173.29±13.19
*t value*	-	-	24.686	34.515	32.350
*P value*	-	-	<0.001	<0.001	<0.001

After repeated measurement analysis, the VAS score, UCLA score, CMS score of the two groups were statistically significant in terms of time factors as well as the interaction of grouping and time (P<0.05). LSD-t pairwise comparison showed that there was no statistical difference in the VAS score, UCLA score, CMS score between the two groups before treatment(P>0.05). However, the VAS score of the two groups after treatment were lower than those of the same group before treatment, and the UCLA score and CMS score increased (P<0.05), while the UCLA score and CMS score of the observation group after treatment were higher, and the VAS score was lower (P<0.05).Table-V.

**Table-V:** Comparison of the score values of each item (*χ̅*±*S*, points).

Indicator	Group	n	Before treatment	1 month after treatment	3 months after treatment	F _time point_	F _interaction_	F _between-group_
VAS score	Observation group	65	6.68±1.42	3.86±1.45 ^*^	1.78±0.22 ^*#^	1169.789	18.823	30.887
	Control group	67	6.71±1.37	5.52±1.19 ^*^	2.98±1.12 ^*#^			
	*t value*		0.124	7.199	39.061			
	*P value*		0.902	<0.001	<0.001	<0.001	<0.001	<0.001
UCLA score	Observation group	65	20.12±1.13	25.87±2.31 ^*^	30.98±2.27 ^*#^	4603.879	243.868	66.664
	Control group	67	20.32±1.27	23.45±1.19 ^*^	27.15±1.19 ^*#^			
	*t value*		0.955	7.599	12.192			
	*P value*		0.341	<0.001	<0.001	<0.001	<0.001	<0.001
CMS score	Observation group	65	63.79±3.26	70.38±3.19 ^*^	78.62±4.81 ^*#^	1365.223	89.806	39.036
	Control group	67	63.58±3.51	66.72±3.54 ^*^	72.33±3.67 ^*#^			
	*t value*		0.356	6.234	8.462			
	*P value*		0.722	<0.001	<0.001	<0.001	<0.001	<0.001

***Note:***

* P<0.05 compared with before treatment; #P<0.05 compared with 1 month after treatment.

## DISCUSSION

Analysis of this result shows that the external rotation, abduction, internal rotation and forward flexion of the observation group were better than those of the control group three months after treatment, and the weight-bearing strength of internal rotation, external rotation and forward flexion of the affected limb was higher than that of the control group, demonstrating that arthroscopic triple release combined with rotator cuff repair can better remove loose bodies, release joint adhesion, restore shoulder joint function and expand the joint range of motion. Presumably, the arthroscopic release is considered more precise compared with the traditional operation.^10^ For example, it gives greater full exposure to the internal structure of tissue, making collateral injury at risk mitigation.

For the triple release, it can get the first release of the subscapular tendon to fully release fibrous adhesion of shoulder joint, and then bring a 360° release of the articular capsule-glenoid labrum ligament complex, which is beneficial for improving the internal and external rotation and abduction motion of the shoulder joint.^11^,^12^ What’s more, if the rotator cuff repair is combined, the continuity and integrity of the rotator cuff structure can be restored, and the rotation of the shoulder joint and humerus can be stabilized to maintain the balance of muscle strength and reduce the likelihood of rotator cuff re-tearing.^13^,^14^

At the same time, some researchers^15^ consider that the primary lesion in the frozen shoulder lies in the rotator interval, where there are a large number of inflammatory factors, which worsen the current pain. In this regard, it is necessary to assess the level of serum pain mediums postoperatively to figure out the recovery of the disease. Among many pain mediums, β-EP, an opioid peptide neurotransmitter, can alleviate physical pain and regulate nociceptors. PGE2 and SP are neuropeptides that can regulate pain transmission and cause inflammatory lesions.^16^ The results show that the level of β-EP was higher and that of PGE2 and SP were lower in the observation group after treatment, indicating that arthroscopic triple release combined with rotator cuff repair could reduce the inflammatory reaction of local tissues of the shoulder joint, in favor of tissue prognosis.

The reason is speculated that, on the basis of rotator cuff repair, combined release can better release the glenohumeral ligament, eliminate the inflammatory tissue in the rotator interval, and completely remove the subacromial bursal tissue to facilitate the recovery of postoperative motor function^17^ Besides, the UCLA score, CMS score and VAS score in the observation group were higher after treatment, indicating that the combined operation could effectively and thoroughly release the adhesion around the shoulder joint, relieve local pain, correct the biomechanical structure of the shoulder joint and promote the functional recovery of the shoulder joint.^18^

### Limitations of the study:

It includes no long-term follow-up was performed and a limited sample size was included. In this regard, corresponding measures should be taken in the future to improve this study in order to further explore the efficacy of this surgical procedure.

## CONCLUSIONS

To sum up, for patients with rotator cuff injury and shoulder freezing, the triple release combined with rotator cuff repair under arthroscopy has affirmative effect, it with less trauma and high safety, which can effectively relieve shoulder joint adhesion, relieve body pain, and improve shoulder joint function.

### Authors’ Contributions:

**JH** and **LC:** Designed this study, prepared this manuscript, are responsible and accountable for the accuracy and integrity of the work.

**KW:** Collected and analyzed clinical data.

**YX:** Data analysis, significantly revised this manuscript.
